# Ongoing Pregnancy Rates in Women with Low and Extremely Low AMH Levels. A Multivariate Analysis of 769 Cycles

**DOI:** 10.1371/journal.pone.0081629

**Published:** 2013-12-16

**Authors:** Alon Kedem, Jigal Haas, Liat Lerner Geva, Gil Yerushalmi, Yinon Gilboa, Hanna Kanety, Mirit Hanochi, Ettie Maman, Ariel Hourvitz

**Affiliations:** 1 IVF unit, Department of Obstetrics and Gynecology, Sheba Medical Center, Tel Hashomer, affiliated with the Sackler Faculty of Medicine, Tel Aviv University, Tel Aviv, Israel; 2 Women and Children's Health Research Unit, Gertner Institute for Epidemiology and Health Policy Research Ltd, Tel Hashomer, affiliated with the Sackler Faculty of Medicine, Tel Aviv University, Tel Aviv, Israel; 3 Institute of Endocrinology, Sheba Medical Center, Tel Hashomer, affiliated with the Sackler Faculty of Medicine, Tel-Aviv University, Tel-Aviv, Israel; Baylor College of Medicine, United States of America

## Abstract

**Background:**

The ideal test for ovarian reserve should permit the identification of women who have no real chance of pregnancy with IVF treatments consequent upon an extremely reduced ovarian reserve. The aim of the current study was to evaluate pregnancy rates in patients with low AMH levels (0.2–1 ng/ml) and extremely low AMH levels (<0.2 ng/ml) and to determine the cumulative pregnancy rates following consecutive IVF treatments.

**Methods:**

We conducted an historical cohort analysis at a tertiary medical center. Serum AMH levels were measured at initial clinic visit and prior to all following treatment cycles in 181 women (769 cycles) with an initial AMH level ≤1 ng/ml, undergoing IVF-ICSI. Main outcome measures were laboratory outcomes and pregnancy rates.

**Results:**

Seventy patients undergoing 249 cycles had extremely low AMH levels (≤0.2 ng/ml), whereas 111 patients undergoing 520 cycles had low AMH levels (0.21–1.0 ng/ml). Number of oocytes retrieved per cycle, fertilized oocytes and number of transferred embryos were significantly lower in the extremely low AMH levels group compared to the low AMH levels (P<0.003). Crude ongoing pregnancy rates were 4.4% for both groups of patients. Among 48 cycles of women aged ≥42 with AMH levels of ≤0.2 ng/ml no pregnancies were observed. But, in patients with AMH levels of 0.2–1.0 ng/ml, 3 ongoing pregnancies out of 192 cycles (1.6%) were observed. However, in a multivariate regression analysis adjusted for age and cycle characteristics, no significant differences in ongoing pregnancy rates per cycle between the two groups were evident. Cumulative pregnancy rates of 20% were observed following five cycles, for both groups of patients.

**Conclusions:**

Patients with extremely low AMH measurements have reasonable and similar pregnancy rates as patients with low AMH. Therefore, AMH should not be used as the criterion to exclude couples from performing additional IVF treatments.

## Introduction

One of the most challenging topics in fertility treatment is the decision concerning those patients who should be denied further treatment and who require referral to an egg donation program, especially in an environment where fertility treatments are covered by the National Health Service. The ideal test for ovarian reserve should permit the identification of women who have no real chance of pregnancy with IVF treatments consequent upon an extremely reduced ovarian reserve. The exclusion of these couples from assisted reproduction techniques (ART) could effectively reduce costs for the health system. A systematic review assessing predictors for pregnancy based upon ovarian reserve concluded that the accuracy of different ovarian reserve testing is very low [1] limiting its clinical use. Recently, serum levels of anti-Müllerian hormone (AMH) have gained popularity as a measure for ovarian reserve [2], partly because of its intra-cycle stability [3], where it appears superior to FSH in predicting oocyte yield and pregnancy potential in women undergoing ART [4]–[10]. Recently, Weghofer et al. reported in a large retrospective study, reasonable pregnancy and live birth rates in women with AMH levels ranging between 0.1–0.4 ng/ml [11]. By contrast, Nelson and colleagues could not establish any pregnancies in 26 women up to 44 years of age with AMH concentrations ≤0.15 ng/ml using a range of different treatment approaches [12]. Consequently, the counseling and management of women with low AMH levels presents a significant challenge where either cycle cancellation or poor response is anticipated. These couples require protracted treatment programs and should be informed about their probabilities for cycle cancelation, poor response, and low number of embryos for transfer and lower pregnancy rates. Consultation becomes even more difficult following several unsuccessful attempts.

The aim of the current study was to evaluate the pregnancy of patients either presenting with low AMH levels (between 0.2–1 ng/ml) or extremely low AMH levels (≤0.2 ng/ml) and to determine the cumulative pregnancy rates following consecutive IVF treatments in these specific patient groups. It is anticipated that this approach will assist in defining whether extremely low levels of AMH could serve as a marker for withholding fertility treatment.

## Methods

### Ethics statement

An ethics approval was obtained from the Institutional Review Board of Sheba medical center for this retrospective study to be carried out using existing patient data in an anonymous manner. A written consent from individual patients was specifically waived by the approving IRB. The project did not involve any additional intervention or modification from the standard treatment.

### Patients

Between January 2006 and June 2011 we evaluated all 181 women who underwent IVF treatments at the IVF unit Sheba Medical Center and had AMH levels ≤1 ng/ml at initial clinic visit. Exclusion criteria included pregestational diagnostic cases (PGD), and surrogacy.

### AMH measurements

Serum AMH levels were measured at the initial clinic visit and prior to all subsequent treatment cycles. In women who underwent more than one cycle, AMH levels at the initial presentation and prior to the following cycles are reported. The serum was separated and frozen in aliquots at −80°C for future analysis with measurement being performed by enzyme-linked immunosorbent assay (ELISA) according to the manufacturer's instructions [Diagnostic Systems Laboratory, Webster, TX] [13]. The intra- and inter-assay coefficients of variations (CVs) were 4.6 and 8.0%, respectively.

### IVF protocols

The following treatment protocols were included: a long agonist protocol in which ovarian suppression was achieved with a mid-luteal single dose of gonadotropin-releasing hormone (GnRH) analogue (Decapeptyl depot 3.75 mg microcapsules) or a daily dose of Decapeptyl 0.1 mg (Ferring Pharmaceuticals, Israel) or with a daily nasal spray (Suprefact, 600–900 mg/day; Sanofi-Aventis, Thailand). Ovarian stimulation used HMG (Menogon or Menopur; Ferring Pharmaceuticals, Israel) or recombinant FSH (follitropin alpha, Gonal-F, Merck Serono or Follitropin beta, Puregon, Organon Schering Plough) 15 days after verification of complete ovarian suppression. In the antagonist protocol, ovulation induction started on day 3 of the cycle with HMG or recombinant FSH. GnRH antagonist treatment (0.25 mg/day, Cetrorelix, Cetrotide, Merck Serono or Ganirelix, Orgalutran 0.25 mg/day, Organon Schering Plough) was started when a follicle of 13 mm was present. In the flare-up protocol, daily Decapeptyl 0.1 mg was initiated on day 2 of menstruation and followed by ovarian stimulation with HMG or recombinant FSH. The initial dose of ovarian stimulation was dependent upon patient age, body mass index and prior treatment history. When three leading follicles reached 18 mm in diameter, patients received human chorionic gonadotropin (Ovitrelle 250 µg; Merck Serono). Oocyte retrieval was performed with transvaginal ultrasound-guided needle aspiration. The ongoing pregnancy rate was defined as the number of pregnancies confirmed by ultrasound scan and continued for at least 21 weeks after embryo transfer. Cycle cancellation criteria: A failure to obtain a minimum of one mature follicle (16–20 mm. This criteria was applied for all patients.

### Statistical analysis

Statistical analysis was performed using SAS statistical software (version 9.1, SAS Institute, Inc., Cary, NC). Categorical variables were expressed as percentages and continuous variables were expressed as means ± SD or as medians and ranges. Comparison of variables was performed with the Chi-square test for categorical variables or the Wilcoxon rank sum test for continuous variables. All tests were 2 sided with P values <0.05 being considered statistically significant. The SAS procedural PHREG was used to calculate adjusted success rates with hazard ratios (HR) and appropriate plus 95% confidence intervals (95%CI). Because of the discrete time scale (i.e. cycle), a logistic regression model was utilized with age at treatment, the number of embryos transferred and the number of fresh or frozen embryos being included in the model as cycle-dependent variables. Adjustments were made for the etiology of infertility (male/female) and the initial AMH levels. Cumulative ongoing pregnancy rates were calculated using Kaplan- Meier life-table analysis

## Results

### Patients

A total of 181 women undergoing 769 IVF-ICSI cycles were included in the analysis. The women were divided into two groups according to their AMH levels including 70 patients (249 cycles) with extremely low AMH levels (≤0.2 ng/ml; mean 0.11±0.05) and 111 patients (520 cycles) with low AMH levels (0.2–1.0 ng/ml; mean 0.5±0.2). *Table 1* summarizes patient demographic characteristics for different AMH groups. There were no significant differences with regards to patient's age at first cycle, type of infertility, etiology of infertility and total gonadotropin dosage.

**Table 1:** Patients characteristics of 181 women with low serum AMH levels (0.2–1 ng/ml) and extremely low serum AMH levels (≤0.2 ng/ml).

	Extremely low AMH levels (≤0.2 ng/ml)	Low AMH levels (0.2–1 ng/ml)
No. of patients	70	111
**Age group(years; n, %)**		
19–35	20 (28.6)	28 (25.5)
36–40	29 (41.4)	34 (30.9)
41+	21 (30.0)	48 (43.6)
Median	38	38
**Etiology (n, %)**		
male	17 (24.3)	28 (25.2)
female	46 (65.7)	69 (62.2)
unknown	7 (10.0)	14 (12.6)
**Total gonadotrophin dosage (I.U) (mean±SD)**	3296±2032	3222±1600

### Cycle characteristics and pregnancy rates

Cycles characteristics are presented in *Table 2*. The group with extremely low levels of AMH had an average retrieval of 3.2±2.7 oocytes per cycle with 65% of the cycles producing 3 or less oocytes per cycle, as compared to 4.8±3.4 oocytes retrieved per cycle in the group with low AMH levels where 42% of the cycles produced 3 or less oocytes per cycle. However, only 8% of the cycles in those with extremely low AMH levels had 5 or more fertilized oocytes per cycle versus 25% in cycles of patients with a low AMH. The fertilization rate was similar between the groups (68% and 66% respectively). Overall, 115 cycles (22%) produced no embryo for transfer in the low AMH group as compared with 81 cycles (32%) where no embryo was available for transfer in the extremely low AMH group (P<0.001). The average number of embryos for transfer was 1.9±0.9 and 2.2±0.9, respectively for the 2 groups (P<0.0001). Nevertheless, there were no significant differences in the clinical and ongoing pregnancy rates per cycle between the extremely low and the low AMH groups (6.8% and 4.4% vs. 7.1% and 4.4%, respectively) or in the clinical and ongoing pregnancy rates per patient (23% and 16% vs. 30% and 20%, respectively).

**Table 2:** 769 cycles parameters in 181 women with low serum AMH levels (0.2–1 ng/ml) and extremely low serum AMH levels (≤0.2 ng/ml).

	Extremely low AMH levels (≤0.2 ng/ml)	Low AMH levels (0.2–1 ng/ml)	P
Cycles	N = 249	N = 520	
**Oocytes retrieved per cycle**			
Mean ±SD	3.2±2.7	4.8±3.4	<0.0001
Median (range)	2 (0–14)	4 (0–21)	
**Fertilized oocytes**			
mean ±SD	2.2±1.6	3.2±2.2	<0.0001
Median (range)	2 (0–10)	3 (0–16)	
**Mean No. of embryos (SD)**	1.9±0.9	2.2±0.9	<0.0001
Median (range)	2 (1–6)	2 (1–5)	
Fresh embryos (No. of cycles, %)	236(95)	457(88)	<0.003
Frozen embryos (No. of cycles, %)	13(5.2)	63(12)	<0.003
**Pregnancy rates per cycle**	**No. of cycle (%)**	**No. of cycle (%)**	
Clinical Pregnancies per cycle	17(6.8)	37(7.1)	NS
Ongoing pregnancies per cycle	11(4.4)	23(4.4)	NS
**Pregnancy rates per patient**			
Clinical Pregnancies per patient	16(22.9%)	34(30.6%)	NS
Ongoing pregnancies per patient	11(15.7%)	23(20.7%)	NS

We couldn't observe any significant differences in pregnancy outcomes between different stimulation protocols.

### Multivariable regression analysis of ongoing pregnancy rates

*Table 3* summarizes the results of a multivariate regression analysis of the ongoing pregnancy rates adjusted for patient's age, the etiology of infertility, the number of transferred embryos and the number of fresh or frozen embryos. Cycles in those with extremely low AMH levels had a 4.4% ongoing pregnancy rate per cycle, which was the same rate observed in those with low AMH levels (adjusted success ratio 1.12; 95% CI 0.51–2.48, P<0.77). However, patients aged 41 years or older had a significantly lower ongoing pregnancy rate than the younger patients (adjusted success ratio 0.23; 95% CI 0.08–0.63, P<0.004). Further, ongoing pregnancy rates were significantly higher in patients who had multiple embryo transfers when compared with those patients with a single embryo transfer (adjusted success ratio 4.37; 95% CI 1.81–10.5, P<0.001). The transfer of frozen or fresh embryos and the underlying etiology of infertility did not significantly influence the ongoing pregnancy rates within the groups.

**Table 3:** Multivariable analysis of ongoing pregnancy rates (34 ongoing pregnancies in 769 cycles, 181 women).

Variables	No. Cycles	Ongoing pregnanciesn (%)	Adjusted success ratio (95% C.I)	p-value
**AMH (ng/ml)**				
≤0.2	249	11(4.4)	1.12 (0.51–2.48)	0.77
0.2–1	520	23(4.4)	Reference	
**Age (Y)**				
19–35	168	12 (7.1)	Reference	
36–40	227	15 (6.6)	0.98 (0.43–2.21)	0.95
41+	374	7 (1.9)	0.23 (0.08–0.63)	0.004
**Etiology**				
male	182	11 (6.0)	Reference	
female	553	20 (3.6)	1.09 (0.48–2.48)	0.83
unknown	34	3 (9.1)	1.80 (0.42–7.68)	0.43
**No. of Transferred Embryos**				
1	368	7 (1.9)	Reference	
2+	400	27 (6.8)	4.37 (1.81–10.5)	0.001
**Fresh vs Frozen embryos**				
Fresh embryos	692	28 (4.0)	Reference	
Frozen embryos	76	6 (7.9)	2.46 (0.89–6.79)	0.08

### Pregnancy rates per age

Analysis of pregnancy rates in patients younger or older then 42 years of age is shown in *Table 4*. We were unable to demonstrate any ongoing pregnancy amongst 48 cycles of 18 patients aged ≥42 years of age with AMH levels ≤0.2 ng/ml. Women at the same age but with AMH levels between 1–0.2 ng/ml had 3 ongoing pregnancies in 192 cycles (1.6%). Moreover, patients younger than 42 years of age had a significantly higher clinical and ongoing pregnancy rates per cycle and per patient than the older group, although clinical and ongoing pregnancy rates per cycle and per patient were not statically different between patients with low AMH as compared to those with an extremely low AMH level.

**Table 4:** Analysis of pregnancy rates per age.

	<Age 42 years (n = 127) women			≥ Age 42 years (n = 54) women		
	Extremely low AMH levels (≤0.2 ng/ml)	Low AMH levels (0.21–1 ng/ml)	P	Extremely low AMH levels (≤0.2 ng/ml)	Low AMH levels (0.2–1 ng/ml)	P
Clinical pregnancies per cycle	15/201(7.5)	23/328(7)	0.74	2/48(4)	10/192(5)	0.77
Ongoing pregnancies per cycle	11/201(5.5)	20/328(6)	0.75	0/48(0)	3/192(1.6)	0.99
Clinical pregnancies per patient	14/52(27)	25/75(33)	0.37	2/18(11.8)	9/36(25)	0.47
Ongoing pregnancies per patient	11/52(21)	20/75(27)	0.42	0/18(0)	3/36(8.3)	0.54

### Cumulative pregnancy rates

**Figure 1:**
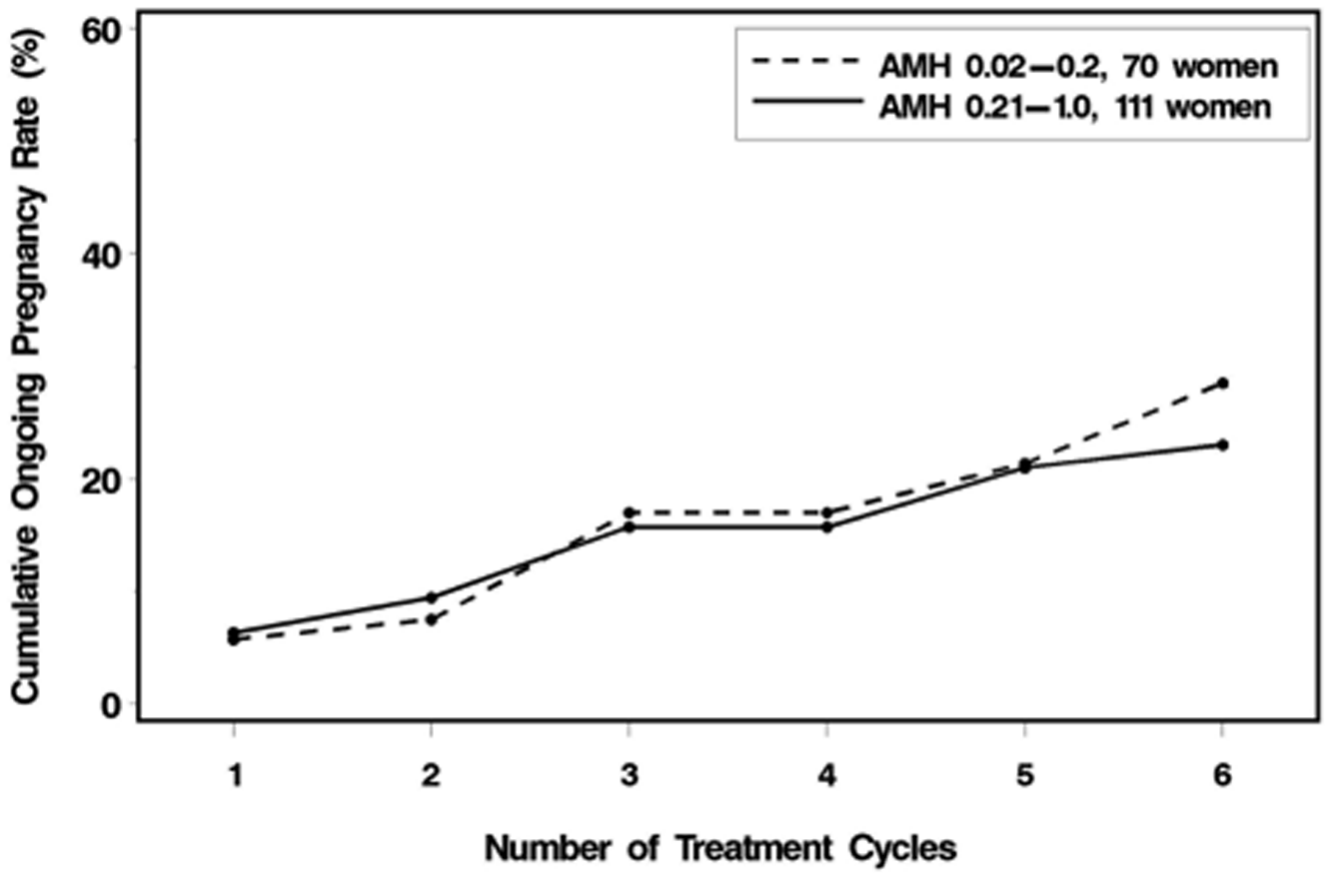
Cumulative ongoing pregnancy rates for patients with AMH level of 0.2–1 ng/ml or ≤0.2 ng/ml.

*Figure 1* shows the cumulative pregnancy rates for patients with low AMH levels (n = 111) and with extremely low AMH levels (n = 70). Analysis of the cumulative pregnancy rates revealed that after 5 cycles, this rate reached 20% for both groups of patients and after 6 cycles the rates were 29% and 22%, respectively.

## Discussion

This large historical cohort study assessing patients presenting with an AMH level lower than 1 ng/ml, shows an association between AMH and all quantitative parameters but with no differences in pregnancy rates between patients with low or extremely low AMH levels throughout all age ranges. Patients with extremely low AMH levels (≤0.2 ng/ml) had low but reasonable ongoing pregnancy rates where it appears that they may benefit from consecutive IVF cycles.

As far as we are aware, this is the largest available study examining ongoing pregnancy rates in patients with AMH levels of ≤0.2 ng/ml (249 cycles). As in Israel, IVF cycles are fully covered by the National Health Service insurance, there are no financial constraints for patients who discontinue treatment, eliminating selection bias within the study population. This ensures that there is almost no patient withdrawal through several treatment attempts [14], permitting a better estimate of the cumulative pregnancy rates in consecutive cycles in this population.

In an attempt to discover an AMH threshold, which would identify women who have a chance of pregnancy after IVF, which is close to zero, we chose an AMH level of ≤0.2 ng/ml that is considered to be extremely low. Our results show that women with AMH levels of ≤0.2 ng/ml still had 4.4% ongoing pregnancy rates per cycle and 16% ongoing pregnancy rates per patient. It is suggested that this cohort can benefit from consecutive IVF cycles, achieving 22% cumulative ongoing pregnancy rates after 6 cycles, however, within this group, those patients ≥42 years of age had no ongoing pregnancies. This data should be viewed with caution due to the limited size of the patient subgroup (48 cycles). Our data concur with that of Nelson et al. who reported reduced but not negligible pregnancy potential in women with AMH levels of 0.7 ng/ml [12] as well as with data from Weghofer and Dietrich [11] showing that women with AMH levels of ≤0.4 still had 6.3% delivery rates per cycle with 1.7% deliveries per cycle for patients >42 years of age and with Bhide et al. who failed to establish a cut-off concentration of AMH below which there were no clinical pregnancies [15]. This shows that the ongoing pregnancy rate is reasonable even in this group of patients.

AMH has become a prominent tool for the evaluation of ovarian reserve. Recent studies have shown a strong correlation between AMH levels and pregnancy rates [9], [12], [16]–[18] where it is expected that the lower the AMH level, the lower the pregnancy rate. This data is confounded by studies, which have included a wide range of AMH levels, rather than a population limited to the lower end of the AMH spectrum. The second aim of this study was to examine the difference in IVF outcomes in patients with low (1–0.2 ng/ml) and extremely low (≤0.2 ng/ml) AMH levels. As expected, there was a lower number of oocytes retrieved, a lower number of fertilized oocytes and a lower number of transferred embryos in cycle of patients with extremely low AMH levels, however, there was no significant difference in any of the pregnancy rates. Our data indicates that in its lower range, AMH remains a good quantitative marker of the ovarian follicular pool but is a poor qualitative marker. This is consistent with previous reports [19]–[25] where in the group of patients with very low ovarian reserve age has a major impact on the chance of pregnancy. This most probably reflects the quality of the oocyte.

AMH is still one of the best available tool for the detection of low ovarian reserve and its role as a clinical test is clear. However, it also appears that reducing the AMH cut-off level below 1 ng/ml by itself does not improve the predictive capacity of pregnancy occurrence following IVF. Therefore, extremely low cut-off AMH values should not be used to exclude couples from IVF treatment. Our data may assist physicians in encouraging patients younger than 42 years of age who present with extremely low AMH levels to continue to pursue treatment after failed IVF procedures.
